# S9.6-based hybrid capture immunoassay for pathogen detection

**DOI:** 10.1038/s41598-023-49881-w

**Published:** 2023-12-19

**Authors:** Ankur Bothra, Megan L. Perry, Elena Wei, Mahtab Moayeri, Qian Ma, Marco A. Biamonte, Marina Siirin, Stephen H. Leppla

**Affiliations:** 1 Microbial Pathogenesis Section, Laboratory of Parasitic Diseases, National Institute of Allergy and Infectious Diseases, Bethesda, MD USA; 2 Drugs and Diagnostics for Tropical Diseases, San Diego, CA USA

**Keywords:** Biological techniques, Microbiology, Molecular biology

## Abstract

The detection of pathogens is critical for clinical diagnosis and public health surveillance. Detection is usually done with nucleic acid-based tests (NATs) and rapid antigen tests (e.g., lateral flow assays [LFAs]). Although NATs are more sensitive and specific, their use is often limited in resource-poor settings due to specialized requirements. To address this limitation, we developed a rapid DNA-RNA Hybrid Capture immunoassay (HC) that specifically detects RNA from pathogens. This assay utilizes a unique monoclonal antibody, S9.6, which binds DNA-RNA hybrids. Biotinylated single-stranded DNA probes are hybridized to target RNAs, followed by hybrid capture on streptavidin and detection with S9.6. The HC-ELISA assay can detect as few as 10^4^ RNA molecules that are 2.2 kb in length. We also adapted this assay into a LFA format, where captured *Bacillus anthracis rpoB* RNA of 3.5 kb length was detectable from a bacterial load equivalent to 10^7^ CFU per 100 mg of mouse tissue using either HC-ELISA or HC-LFA. Importantly, we also demonstrated the versatility of HC by detecting other pathogens, including SARS-CoV-2 and *Toxoplasma gondii*, showing its potential for broad pathogen detection. Notably, HC does not require amplification of the target nucleic acid and utilizes economical formats like ELISA and LFA, making it suitable for use in sentinel labs for pathogen detection or as a molecular tool in basic research laboratories. Our study highlights the potential of HC as a sensitive and versatile method for RNA-based pathogen detection.

## Introduction

The accurate identification of the pathogen responsible for an infection is essential for effective treatment and control of infections. Currently, most molecular diagnostics for pathogen identification rely on antigen detection tests or nucleic acid-based tests (NATs). NATs are widely used in clinical and field settings to diagnose viral, bacterial, fungal, protozoan, and helminth infections in humans, plants, and animals by detecting pathogen-specific genetic material, including DNA, RNA, or both^1–4^. For example, PCR tests are widely used for the detection of SARS-CoV-2 RNA. Various molecular techniques that identify single or multiple genes from pathogens, including those from SARS-CoV-2, include quantitative real-time PCR (qPCR), droplet digital PCR (ddPCR), reverse-transcription loop-mediated isothermal amplification (RT-LAMP), CRISPR-Cas13a^5^, next-generation sequencing (NGS), and micro-NMR (µNMR). Although NATs have high sensitivity and specificity, they are limited by the requirement for amplification of target sequences from the pathogen and specialized laboratory infrastructure, which restricts their use in routine clinical or field settings^6,7^. CRISPR-Cas based NATs do not require sequence amplification and can be used with minimal instrumentation, thereby overcoming the limitations of other NATs. However, CRISPR-Cas based diagnostics may be limited by sensitivity to RNA secondary structures and the need for a protospacer adjacent motif (PAM) sequence in the target genes^5,8–11^.

Low-cost and rapid pathogen tests, such as enzyme-linked immunosorbent assays (ELISAs) or lateral flow assays (LFAs), have been developed to detect pathogen-specific antigens using capture antibodies^12^. While antigen tests are generally less sensitive than NATs, their affordability and quick results make them a suitable option when resources, cost, and speed are important. However, antigen tests are not ideal as public health tools since they may not detect infections early enough to prevent disease spread. In contrast, NATs can detect organism-specific DNA or RNA sequences in the early stages of an infection before the pathogen load is sufficient for a positive antigen test. In clinical settings, a negative rapid test in a symptomatic individual usually requires a confirmatory NAT, which increases the time to and cost of diagnosis. Thus, there is a need for low-cost, rapid pathogen tests that retain the sensitivity advantages of NATs.

Various low-cost techniques, including ELISA and LFA, have been developed to detect disease-specific nucleic acids^5,10,13–15^. One example is the HC2-HPV diagnostic assay for human papillomavirus (HPV) infection marketed by Qiagen (Digene), which employs polyclonal antibodies in a "hybrid capture" sandwich ELISA format^16,17^. The HC2-HPV assay uses a complementary RNA probe to hybridize with HPV DNA, which is then detected by a polyclonal DNA-RNA duplex-specific antibody. Recently, several methods were developed using DNA-RNA specific antibody for pathogen detection^13–15^. S9.6, a DNA-RNA specific monoclonal antibody, was isolated from mice immunized with a DNA-RNA heteropolymer antigen of bacteriophage ɸX174^18^. Previous studies have shown that S9.6 binds to a 6-bp epitope on DNA-RNA hybrids with high affinity and specificity in a largely sequence-independent manner^18–22^. Furthermore, S9.6 can bind with low affinity to double-stranded RNA (dsRNA)^22–24^.

In this study, we developed a pathogen-specific nucleic acid-based test in both ELISA and LFA formats by utilizing the unique properties of S9.6. Recently, we validated the use of S9.6 Fab in a sandwich ELISA^25^ and used it as a platform to develop our assays in this study. This assay, called DNA-RNA Hybrid Capture Immunoassay (HC), detects pathogen-specific RNA, and can be adjusted to detect any pathogen. The HC assay does not require molecular manipulations such as reverse transcription or pre-amplification of the pathogen's RNA or DNA, nor does it require advanced instrumentation or specialized training to perform. With the HC assay, we could specifically detect RNA for the *pagA*, *rpoB*, and *gyrA* genes of *Bacillus anthracis*, the Spike E gene of SARS-CoV-2, and the B1 gene of *Toxoplasma gondii*. This assay can be used for the detection of pathogens in low-resource settings, as well as for qualitative gene expression analysis.

## Results

### S9.6 specifically binds pre-existing hybrids in total cellular RNA extracts

**Figure 1:**
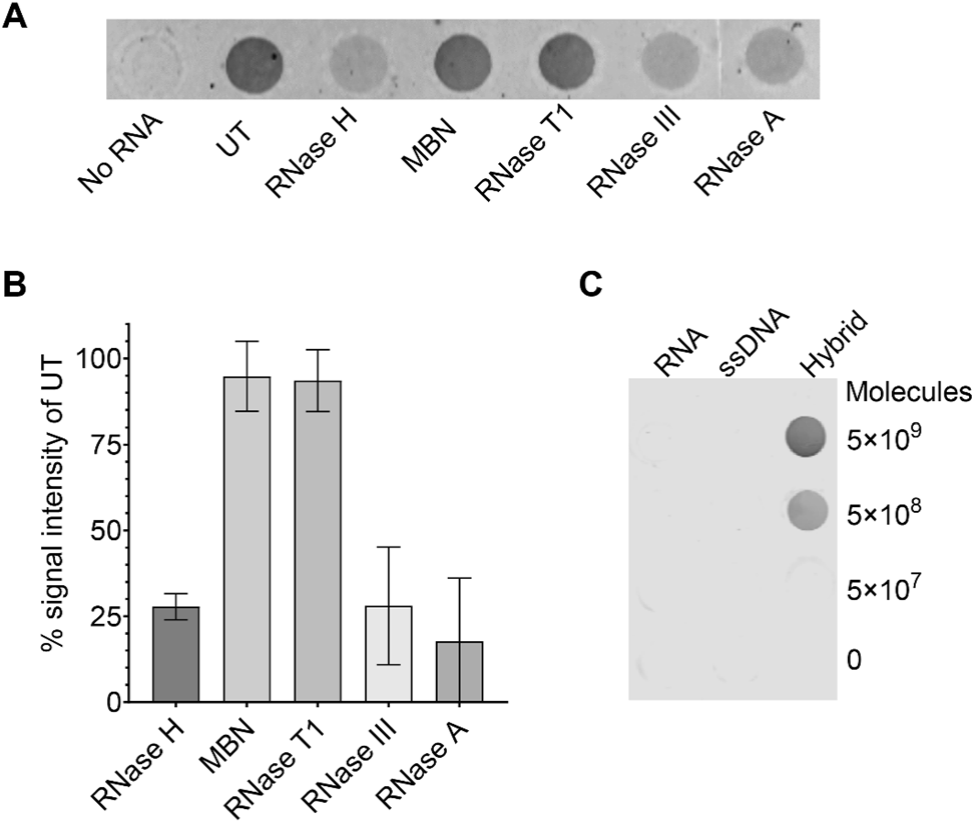
Analysis of S9.6’s specificity for DNA-RNA hybrids. (**A**) Representative dot blot analysis of S9.6 binding to a crude extract of cellular RNA from *B. anthracis*. One microgram of cellular RNA was spotted directly on a nitrocellulose membrane or first treated with respective nucleases. *UT* untreated, *MBN* mung bean nuclease. Nuclease specificities are as follows: RNase H, DNA-RNA hybrids; MBN, ssDNA; RNase T1, ssRNA; RNase III, dsRNA; RNase A, all RNA. (**B**) Quantification of signal intensity relative to untreated (UT) control for nuclease treated samples. Data represents mean ± SD of duplicate dot blots (**A**, Fig. S1A). (**C**) Dot blot analysis using S9.6 to detect in vitro synthesized *pagA* mRNA, ssDNA, and DNA-RNA hybrids in specified amounts.

To develop a specific assay for DNA-RNA hybrids, first, we investigated the nucleic acid species to which S9.6 binds in total cellular RNA extracts (henceforth referred to as “cellular RNA”) since most clinical samples contain a mixture of nucleic acids. Cellular RNA from *B. anthracis* was treated with nucleases of varying specificities, and a dot blot was performed using S9.6 (Fig. 1A). Treatment with RNase H, which selectively degrades RNA in DNA-RNA hybrids, caused a roughly 75% signal loss relative to the untreated control (Fig. 1A,B), confirming that S9.6 binds to pre-existing hybrids present in total nucleic acids. Treatment with the dsRNA-specific nuclease RNase III similarly caused roughly a 75% reduction in signal, suggesting that much of the signal comes from dsRNA despite S9.6's low affinity for dsRNA^19,22–24^.

To determine whether S9.6 binds to ssRNA or ssDNA, we treated cellular RNA with mung bean nuclease and RNAse T1, which specifically digest ssDNA and ssRNA, respectively, and performed a dot blot analysis. We observed no difference in signal, indicating that S9.6 does not bind to ssDNA or ssRNA (Figs. 1A,B, S1A). We further validated this finding by hybridizing in vitro synthesized mRNA from the *B. anthracis* gene *pagA*, a gene encoding protective antigen of anthrax toxin, to complementary ssDNA produced using Lambda Exonuclease (Fig. S1B). We performed a dot blot using S9.6 and observed a signal from the hybrid dot, but not from the single-stranded DNA or RNA dots (Fig. 1C). Our results confirm that S9.6 does not bind to the secondary structures of ssDNA or ssRNA, which often form double-stranded structures (Fig. S2A). The limit of detection (LOD) of dot blot analysis for in vitro synthesized *pagA* hybrid was found to be in the sub-nanomolar range (0.82 nM or 1.25 ng/µL), corresponding to 5 × 10^8^ molecules of *pagA* DNA-RNA hybrid.

### Development of HC-ELISA

**Figure 2:**
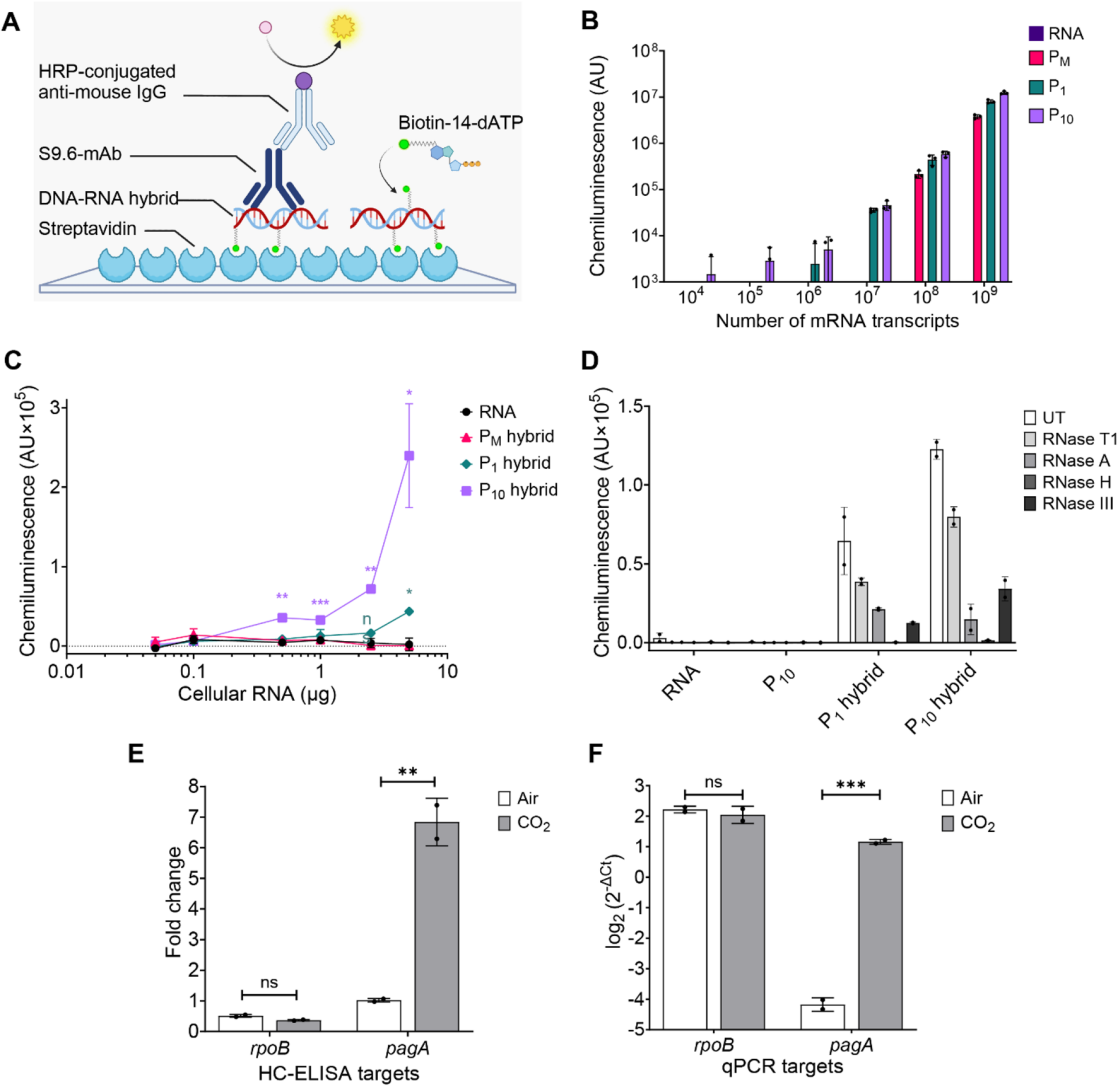
Sensitivity and specificity of HC-ELISA to detect *B. anthracis* gene transcripts*.* (**A**) Schematic of the HC-ELISA format. Gene specific DNA-RNA hybrids are generated using 5’-biotinylated ssDNA probes (P_M_) or 5′-biotinylated ssDNA probes synthesized in the presence of 1 µM (P_1_) or 10 µM (P_10_) biotin-14-dATP. The hybrids are captured on a poly-streptavidin coated ELISA plate and bound to S9.6, which is detected using anti-mouse IgG conjugated with HRP (horseradish peroxidase) and a chemiluminescent substrate. Illustration created with BioRender.com. (**B**) HC-ELISA sensitivity to *pagA* hybrids incorporating P_M_, P_1_, and P_10_ probes. Data represents mean arbitrary units (AU) of chemiluminescence normalized to average of ssDNA and buffer-alone control ± SD (n = 3 wells). (**C**) Signal from HC-ELISAs detecting *rpoB* transcripts in *B. anthracis* cellular RNA. Data represent mean chemiluminescence normalized to average of ssDNA and buffer-alone control ± SD (n = 2 wells). (**D**) Assay specificity assessment using ribonucleases. Data represent mean chemiluminescence normalized to average of ssDNA and buffer-alone control ± SD (n = 2 wells). UT = untreated. (**E**) *rpoB* and *pagA* expression over reference gene *gyrA* between ambient and CO_2_-rich conditions as measured by chemiluminescence from HC-ELISA and (**F**) relative abundance of *rpoB* and *pagA* to *gyrA* as measured by RT-qPCR. Error bars represent mean ± SD of duplicates; statistical significance was determined with Student’s t-test. *p < 0.05, **p < 0.01, ***p < 0.001; ns, not significant. Panels (**B**–**D**) show a representative experiment of three repeated experiments; panels (**E**,**F**) present the data in full.

Our dot blot results confirmed that S9.6 can detect pre-existing hybrids in cellular RNA and may non-specifically bind to highly abundant dsRNA. Therefore, we aimed to develop an assay that excludes dsRNA and pre-existing hybrids by selectively enriching transcripts from cellular RNA. The result was the Hybrid Capture Immunoassay by ELISA (HC-ELISA), which captures biotinylated complementary ssDNA probes hybridized to RNA on a streptavidin-coated surface. The captured hybrids are then detected using S9.6 with chemiluminescence as the signal output (Fig. 2A). To determine the optimal probe type for the assay, HC-ELISA was performed using 5′-mono-biotinylated (hereafter referred to as P_M_) and poly-biotinylated (using 1 µM and 10 µM biotin-14-dATP, hereafter referred to as P_1_ and P_10_) probes hybridized to 2.21-kilobase-long *pagA* RNA synthesized via in vitro transcription. Assuming full hybridization from equal amounts RNA and ssDNA probes (Fig. S2B), the LOD of HC-ELISA for *pagA* RNA hybridized to the P_M_ probe was 10^8^ molecules, while for RNA hybridized to P_1_ and P_10_ probes, it was 10^6^ and 10^4^ molecules, respectively (Fig. 2B). Based on these results, we selected P_1_ and P_10_ probes to maximize the sensitivity of HC-ELISA in further experiments.

### Sensitivity and specificity of HC-ELISA

Observing that HC-ELISA was able to specifically identify DNA-RNA hybrids generated from a pure source of target gene transcripts (in vitro synthesized RNA; Fig. 2B), we next sought to validate the assay’s utility in the detection of target transcripts from a crude source of many gene transcripts since this condition more closely mimics a clinical or field test sample. We also observed that longer hybrids resulted in a higher signal intensity (Fig. S2C); thus, the ~ 3.5 kb mRNA of *B. anthracis* RNA polymerase beta subunit (*rpoB*) was selected as the target transcript in tests of assay sensitivity to hybrids generated in the cellular RNA of *B. anthracis*. P_M_, P_1_, and P_10_ probes (equivalent to ~ 3.5 kb full length gene) were hybridized to *rpoB* transcripts in cellular RNA and assayed by HC-ELISA (Fig. 2C). The P_1_ and P_10_ probes could identify the presence of *rpoB* mRNA in as little as 5 µg and 500 ng of *B. anthracis* cellular RNA, respectively, whereas the P_M_ probe could not enable detection of *rpoB* mRNA in HC-ELISA. The signal to noise ratio (S/N) of the P_10_ probe was found to be fourfold greater than that of the P_1_ probe (Fig. S2D).

To test the specificity of HC-ELISA for detection of longer DNA-RNA hybrids, an array of ribonucleases including RNase H, RNase T1, RNase A, and RNase III was used (Fig. 2D). Samples treated with RNase H, which digests RNA in DNA-RNA hybrids, showed a ~ 85% signal loss in HC-ELISA. In contrast, samples treated with RNase T1, which degrades ssRNA, showed no decrease in signal. Samples treated with RNase III also showed a 65–70% signal loss when compared to untreated controls.

To test the quantitative power of HC-ELISA, the assay was used to evaluate a well-established conditional transcription pattern specific to *B. anthracis.* RT-qPCR and HC-ELISA were performed in parallel using the same extracts of cellular RNA from bacteria grown in either air or CO_2_ (Fig. 2E,F)*.* Gene transcripts *rpoB* and *pagA* were selected as targets and the *gyrA* transcript (encoding gyrase A) was selected as a reference. Under enriched CO_2_ conditions, *pagA* is expressed at levels 130-fold greater than when *B. anthracis* is grown in air, while *rpoB* and *gyrA* are constitutively expressed independent of CO_2_^26^. Hybrid capture signals from *rpoB* and *pagA* were normalized to the signal from *gyrA* to facilitate comparison with qPCR results. HC-ELISA was able to significantly (p > 0.01) identify ~ sixfold induction in the expression of *pagA* mRNA while showing no change in *rpoB* expression in CO_2_ (Fig. 2E) and RT-qPCR indicated a 40-fold induction of *pagA* transcription and no change in *rpoB* transcription under the CO_2_ condition (Fig. 2F).

### HC-ELISA can detect simulated *B. anthracis* infections in mice

**Figure 3:**
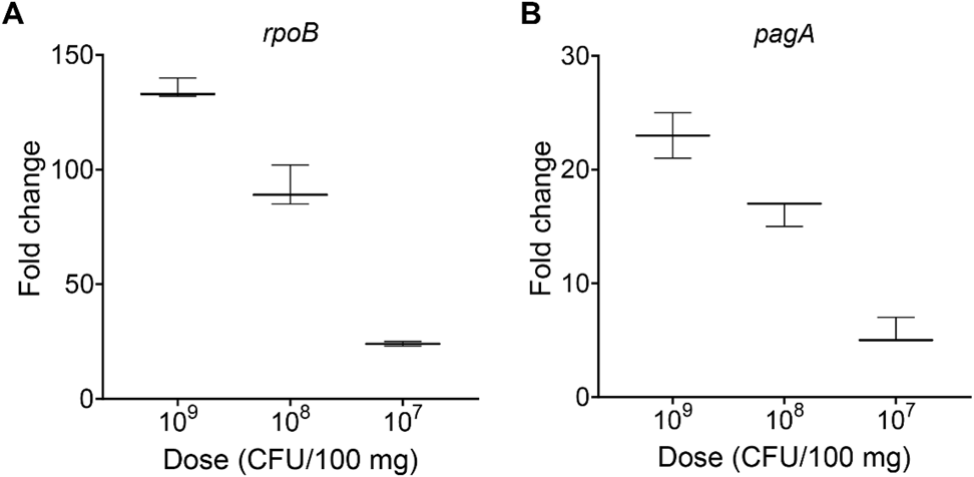
HC-ELISA can detect simulated *B. anthracis* infection in mice. (**A**,**B**) Median fold change in chemiluminescence as a function of bacterial CFU/100 mg of spleen suspension as measured by HC-ELISAs detecting *rpoB* (**A**) and *pagA* (**B**). Fold change was calculated relative to uninfected control spleens. Error bars represent range of triplicate wells in a representative experiment of n = 3.

We observed that the HC-ELISA method is robust in detecting highly expressed gene transcripts from pathogens like *B. anthracis.* To evaluate the assay's performance in detecting simulated infections, *B. anthracis* A35 strain vegetative bacteria were spiked into mouse spleen suspensions at varying bacterial doses ranging from 10^7^ to 10^9^ CFU/100 mg of spleen tissue. The presence of *B. anthracis rpoB* and *pagA* mRNA in cellular RNA extracted from the spleens was then analyzed using HC-ELISA with P_10_ hybridization probes (Fig. 3A,B). As expected, the fold change in chemiluminescence over non-spiked controls was highest in the 10^9^ CFU group and lowest in the 10^7^ CFU group for both genes but *rpoB* showed a higher fold change relative to non-spiked spleen samples. The assay’s efficiency for detecting simulated infection was assessed in comparison to the standard curve generated from assays performed using cellular RNA from bacteria (Fig. S3). For the highest bacterial load (10^9^ CFU/100 mg of spleen), *rpoB* and *pagA* RNA detection in the simulated infection was equivalent to detection from bacteria alone, but efficiency dropped as bacterial load decreased.

### HC-ELISA can be paralleled by a lateral flow assay (HC-LFA)

**Figure 4:**
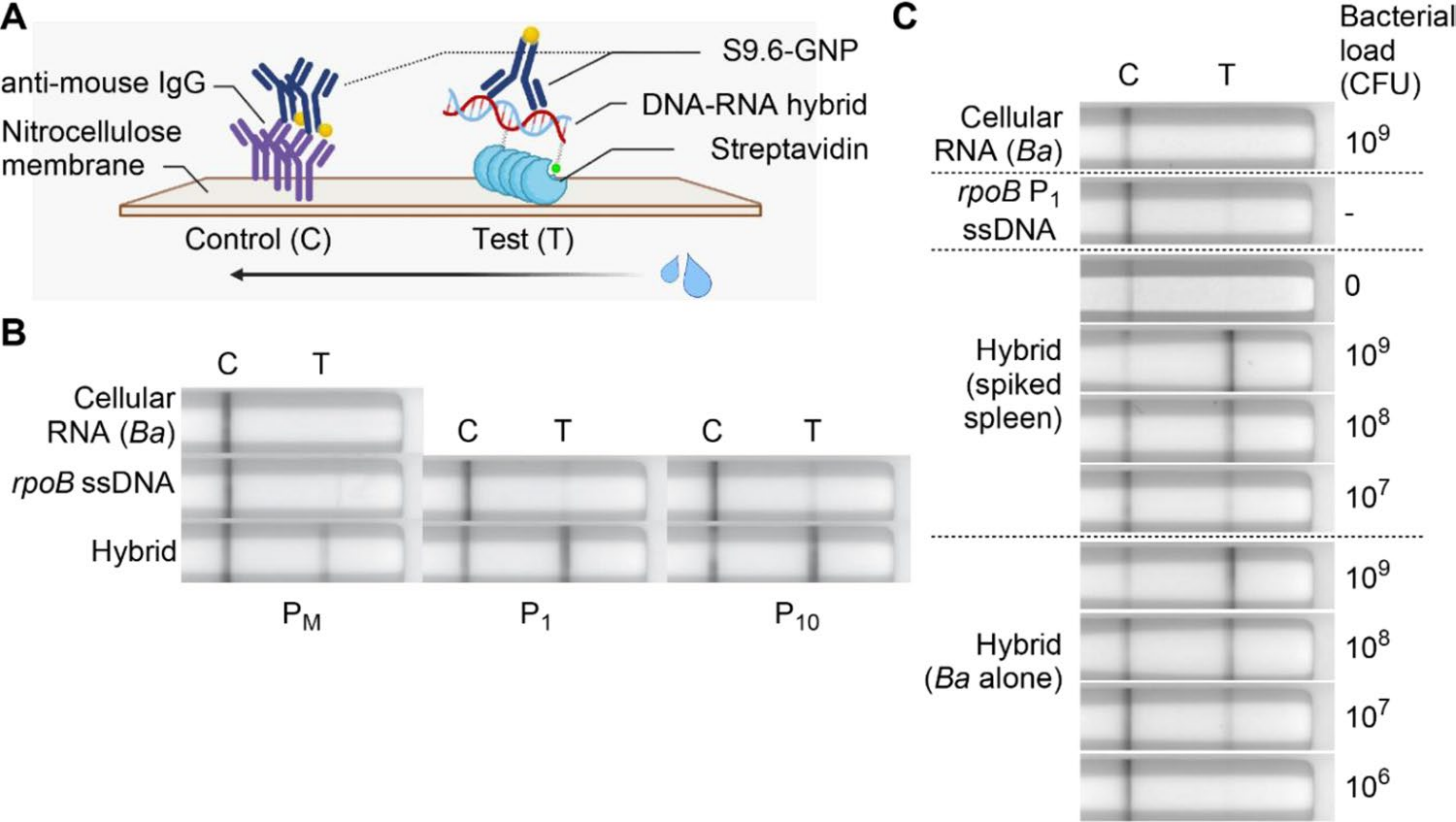
HC-LFA can detect simulated *B. anthracis* infections in mice. (**A**) Schematic of HC-LFA. S9.6-GNP = gold nanoparticle-conjugated S9.6. The arrow indicates the direction of mobile phase. Illustration created with BioRender.com. (**B**) Images of lateral flow assays showing HC-LFA’s specificity for hybrids by probe type (*B. anthracis* RNA, 2 µg). (**C**) Images of lateral flow assays showing HC-LFA to detect *rpoB* transcripts in cellular RNA from *B. anthracis* or *B. anthracis*-spiked murine spleen tissue. The spiked bacterial load ranged from 10^7^ to 10^9^ CFU/100 mg of tissue. For bacteria alone, 2 µg cellular RNA, equivalent to a bacterial load of 10^9^ CFU was diluted. 5 ng of P_1_ probe was used in hybridization and ssDNA controls. Assays were imaged after 30 min. Individual lateral flow strips are placed separately and are grouped within the dashed line in (**B**) and (**C**).

Next, we developed a hybrid capture lateral flow assay (HC-LFA) based on the HC-ELISA method to detect pathogen-specific RNA via DNA-RNA hybrids. The HC-LFA assay involves capturing biotinylated hybrids on a poly-streptavidin test line (T) and detecting them with gold nanoparticle-conjugated S9.6 (S9.6-GNP) (Fig. 4A). The control line (C) in HC-LFA contains anti-mouse IgG, which captures unused S9.6-GNP. We tested P_M_, P_1_, and P_10_ probes hybridized to *rpoB* mRNA in total nucleic acids from *B. anthracis* to determine the best probe for use in HC-LFA. The P_1_ probe provided an optimal balance of test line intensity for true versus false positives and was selected for use in all subsequent lateral flow experiments. False positives of low intensity were observed in assays performed with the *rpoB* P_1_ and P_10_ ssDNA probes alone, but not in assays performed using only cellular RNA (Fig. 4B).

To validate the ability of HC-LFA for *B. anthracis* infection detection, cellular RNA was extracted from spleen suspensions spiked with vegetative *B. anthracis* and used as a source of *rpoB* transcripts for hybridization. Parallel assays were also performed using cellular RNA from bacteria alone to form a standard curve against which to evaluate the assay's efficiency in simulated infections. The assay was positive for hybrids starting at 10^7^ CFU/100 mg of tissue for both spiked spleen and bacteria alone with comparable results at all tested bacterial loads (Fig. 4C, quantifications in Fig. S4A). However, a false positive of low intensity was observed in the assay performed using the *rpoB* P_1_ ssDNA probe alone but not in assays using cellular RNA alone. A 30-min incubation time provided only marginally greater sensitivity than a 20-min incubation, but improved readability with the naked eye when near or at the detection limit of 10^7^ CFU (Figs. 4C, S4A). We next tested the ability to detect *rpoB* RNA in the spleens of mice infected with *B. anthracis* spores and euthanized at various stages of malaise. We found clear detection in 3 of 5 tested samples and less clear detection in one sample (Fig. S4B, Table S1). However, the ability to detect infection was not correlated to malaise grade.

### HC assays can detect RNA from parasite and viral pathogens

**Figure 5:**
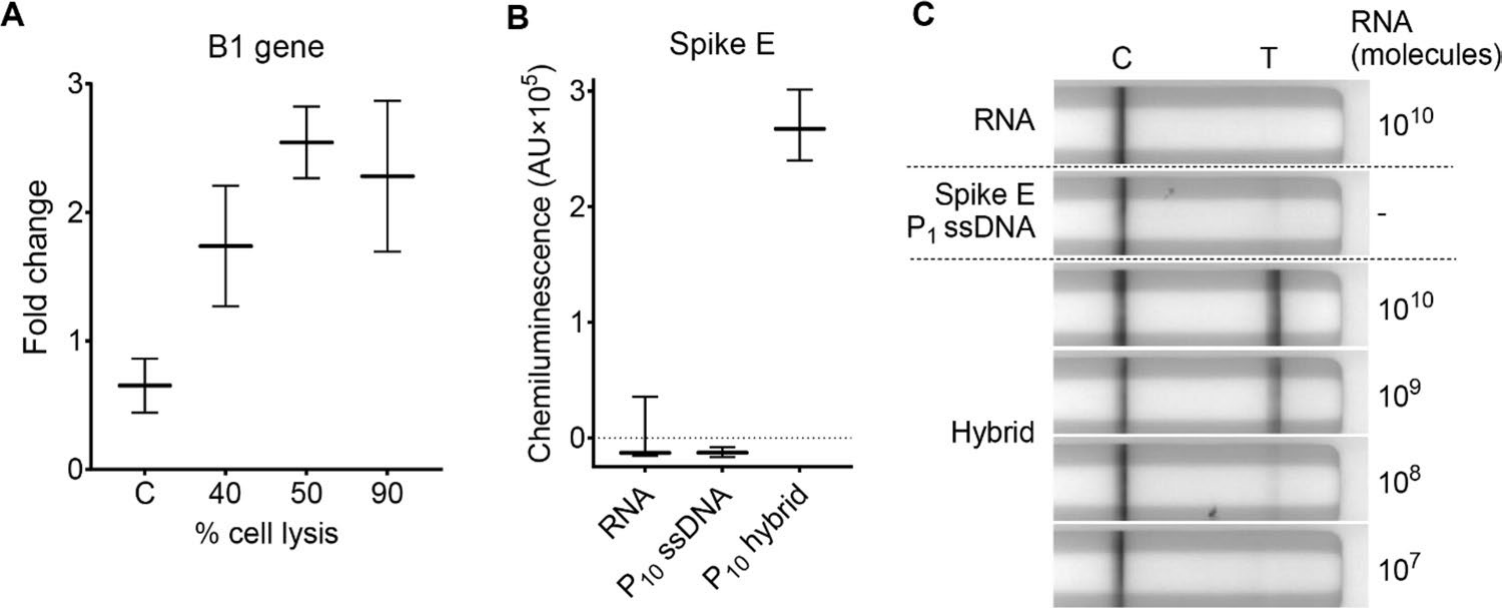
HC assays can detect RNA transcripts from *T. gondii* and SARS-CoV-2. (**A**) Average fold change in chemiluminescence from HC-ELISA to detect B1 gene mRNA of *T. gondii*-infected HFF cells over uninfected cells seeded at the same density at start of study and harvested at the same time as infected cells. The P_10_ probe was used in this experiment and C (control) is the probe hybridized with cellular RNA from overly confluent uninfected cells. Bars represent mean ± SD of duplicate wells in a representative experiment of n = 3. (**B**) Median chemiluminescence from HC-ELISA to detect in vitro synthesized Spike E mRNA of SARS-CoV-2. Data obtained using 2 × 10^9^ molecules/well of specified nucleic acids (assuming full hybridization from equal amounts RNA and probe for P_10_ hybrid). Bars represent range of triplicate wells in a representative experiment of n = 3. (**C**) Lateral flow assays show the sensitivity of HC-LFA to Spike E gene hybrids generated from 5 ng (2.6 × 10^9^ molecules) P_1_ probe and in vitro-synthesized mRNA in amounts specified. Assays were imaged after 30 min. Individual lateral flow strips are placed separately and are grouped within the dashed line.

After demonstrating the effectiveness of HC assays in detecting gene-specific transcripts from bacteria, we aimed to extend the assay's applicability to diverse pathogens, such as *Toxoplasma gondii* (strain Prugniaud (PRU)) and SARS-CoV-2. To assess HC-ELISA’s ability to detect the presence of *T. gondii*, P_10_ probe (~ 2.2 kb) was hybridized to transcripts of the highly repeated B1 gene in cellular RNA extracted from infected human foreskin fibroblasts (HFF)^27^. Figure 5A shows that HC-ELISA could specifically identify B1 gene RNA from *T. gondii*-infected HFF cells when approximately 40% of the monolayer was lysed. The fold change in signal increased at higher parasite loads (monitored visually) indicated by the increase in percent cell lysis; however, an increase in cell lysis from 50 to 90% did not lead to further increase in signal over uninfected controls. We also assayed cellular RNA from overly confluent uninfected cells (C) to assess the potential for non-specific probe binding. No non-specific detection of transcript was seen in over-confluent HFF cells.

Next, we assessed HC-ELISA and HC-LFA for their ability to detect SARS-CoV-2 Spike E (also known as *S*) gene RNA. Spike E mRNA was synthesized by in vitro transcription and hybridized to an equal number of P_10_ probe (~ 3.7 kb) molecules for detection in HC-ELISA. HC-ELISA could clearly detect Spike E transcripts at 2 × 10^9^ molecules of P_10_ hybrid (Fig. 5B). For HC-LFA, hybrids were produced using 5 ng (2.6 × 10^9^ molecules) P_1_ probe and transcript abundance ranged from 0.02 to 20 ng (10^7^ to 10^10^ molecules); the assay was able to detect down to 10^8^ molecules of Spike E RNA with no false positives visible on the RNA strip and a minimal false positive visible on the ssDNA probe strip (Fig. 5C).

## Discussion

The Hybrid Capture immunoassay (HC) developed in this study utilizes the DNA-RNA binding properties of S9.6 to specifically capture and detect pathogen-specific RNA transcripts without requiring sequence amplification. This assay was successful in identifying mRNA from known pathogens such as *B. anthracis*, *T. gondii*, and SARS-CoV-2, demonstrating its pan-pathogen adaptability.

S9.6 has been extensively utilized in recent years for investigating R-loop metabolism (single-stranded genomic DNA annealed to nascent mRNA) through techniques like DRIP-Seq (DNA-RNA immunoprecipitation followed by high-throughput DNA sequencing) and DRIPc-Seq (DNA-RNA immunoprecipitation followed by cDNA conversion coupled to high-throughput sequencing)^28–31^. Prior research has employed S9.6 for whole cell-based R-loop identification; however, since S9.6 displays some affinity for dsRNA^24^, the quality of results varies across studies, whether using immunofluorescence^32–36^ or dot blots^37^. In our dot blots, dsRNA-specific nuclease RNase III treatment showed that some signal from S9.6 bound to *B. anthracis* cellular RNA originated from dsRNA. Furthermore, treatment with RNase H—specific for RNA in DNA-RNA hybrids—revealed that approximately half of the signal stemmed from pre-existing hybrids in the form of R-loops. The dsRNA signal may be due to S9.6 detection of bacterial ribosomal RNA, which constitutes over 60% of a cell's total nucleic acid content^24^. Interestingly, samples containing only in vitro synthesized RNA showed no signal even with extensive double-stranded secondary structures predicted by mFold^23^. Any assay which uses S9.6 to detect pathogen transcripts hybridized to DNA probes in a cellular RNA sample must be designed to overcome the challenges posed by both pre-existing hybrids and competition from abundant dsRNA.

Recognizing the potential utility of this antibody beyond R-loop detection, we utilized the biotin-streptavidin interaction to enrich specific mRNA transcripts. Biotinylated ssDNA probes anneal to their RNA counterparts, which are then selectively captured on the streptavidin-coated plate surface. We assessed three types of biotinylated ssDNA probes for their impact on the sensitivity of HC assays. Mono-biotinylation in P_M_ probes was accomplished by directly using 5′-biotinylated primers. However, these probes displayed suboptimal performance in both HC-ELISA and HC-LFA assays likely because of the low amount of biotin per probe. Previous studies suggested that Taq polymerase's 5′ flap endonuclease activity enables the incorporation of modified deoxyribonucleotides, such as biotin-14-dATP, into DNA strands via nick-translation^38,39^. We hypothesized that the incorporation of biotin-14-dATP would sufficiently biotinylate probes to enable sensitive detection of probe-target hybrids in HC assays. Indeed, the use of P_1_ and P_10_ *pagA* probes proved successful, facilitating the detection of *pagA* transcripts from 10^6^ and 10^4^ molecules, respectively.

Our primary selection criterion for target transcripts from each pathogen was specificity; secondary considerations included abundance and length. Transcripts encoding the protective antigen (PA) toxin (*pagA*, 2.2 kb), whose expression is induced by CO_2_^26^, and AtxA (*atxA*, 0.8 kb), a transcription factor that regulates virulence genes including *pagA,* are unique to *B. anthracis* and provide a high degree of pathogen specificity on this basis. Transcripts encoding RNA polymerase beta subunit (*rpoB*, 3.5 kb) and gyrase A (*gyrA*, 2.5 kb) are constitutively expressed and are found in all bacteria, with *rpoB* displaying sequence variation which enables distinction even between members of *Bacillus*^40^. Similarly, the B1 gene of *T. gondii* is established as specific for the parasite and its multicopy locus is transcribed in tandem to produce 1.3 kb-long spliced transcripts, which were of sufficient length for our assay^27^. Lastly, transcripts of the Spike E (also known as *S*) gene (3.7 kb) encoding the spike protein from SARS-CoV-2 provided assay specificity to this virus.

After establishing that HC-ELISA could specifically detect hybrids from in vitro-synthesized *pagA* mRNA, we assessed its capability to identify hybrids generated from total bacterial RNA. Given S9.6’s largely sequence-independent 6-bp epitope, we reasoned that longer hybrids would enhance assay sensitivity and thus chose to detect *rpoB* in cellular RNA; *rpoB* is longer (3.5 kb) than *pagA* (2.2 kb), providing a larger number of S9.6 epitopes per hybrid. As observed with *pagA*, HC-ELISA using P_1_ and P_10_ *rpoB* probes generated signal above the cellular RNA background.

With the limit of detection (LOD) for HC-ELISA ranging from 10^4^ to 10^6^ molecules, we inferred that the assay could be employed for semi-quantitative estimation of differentially expressed genes. Several studies have shown that CO_2_ induces *pagA* expression in *B. anthracis* without affecting the expression of constitutive genes like *rpoB* and *gyrA*^26^. Our HC-ELISA results were consistent with these findings, successfully measuring the differential expression of *pagA* between bacteria cultured with and without supplemental CO_2_. To evaluate our assay's performance relative to an established quantitative method, we measured the abundance of *pagA*, *rpoB*, and *gyrA* transcripts in the same samples using RT-qPCR. The results demonstrated a 40-fold increase in *pagA* expression under CO_2_-enriched conditions. In comparison, the sixfold increase detected by HC-ELISA suggests that our assay can effectively identify significant changes in the expression of highly expressed genes.

After establishing the sensitivity and specificity of our HC assay, we then aimed to use the assay to diagnose anthrax infection. Organ infections where vegetative *B. anthracis* was spiked into spleen suspensions at 10^7^ CFU/100 mg were used as a model for anthrax infection. HC-ELISA could detect *B. anthracis rpoB* RNA with a 24-fold greater signal and *pagA* RNA with a fivefold greater signal relative to uninfected controls. Two factors can account for the observed difference in signal intensity between genes: firstly, *rpoB* is a constitutive gene expressed at a higher level than *pagA*, a gene with transcription regulated by various environmental cues. Further, the *rpoB* transcript is also ~ 60% longer, presenting a higher number of epitopes for S9.6 binding.

To demonstrate that the HC format can be employed in low-cost diagnostics, we adapted the HC-ELISA into a lateral flow assay (LFA). Our findings indicate that HC-LFA is sufficiently sensitive to identify pathogen-specific DNA-RNA hybrids in a spiked organ. The assay could also detect *rpoB* transcripts in some but not all spleens of mice infected with *B. anthracis* A35 strain spores. This was not unexpected, as the murine model of anthrax A35 infection of C57BL/6 J mice with 2 × 10^7^ spores rarely yields CFU values exceeding 10^5^ bacteria in pools of whole spleen, liver and kidneys harvested at onset of malaise^41^. Thus, a portion of the spleen from one or two infected animals as used in our studies is unlikely to reach sufficient bacterial counts for detection until terminal stages of disease.

The ability of S9.6 to indiscriminately identify all DNA-RNA hybrids offers pan-pathogen adaptability, which is a significant advantage of HC assays. We successfully detected the presence of *T. gondii* and *B. anthracis* in simulated infection models, as well as SARS-CoV-2 in vitro. However, we observed substantial variability in the limit of detection (LOD) among pathogens and HC formats. The differing LOD between HC-ELISA and HC-LFA can be attributed to the inherent tradeoffs between time, sensitivity, and semi-quantitative detection in HC-ELISA, compared to speed, simplicity, and qualitative detection in HC-LFA. Variability between pathogens, on the other hand, is multifactorial. To achieve maximum sensitivity for a specific pathogen, careful analysis of potential target gene expression characteristics is necessary. This analysis relies on baseline characterization of the pathogen in existing literature, which highlights the assay's primary limitation: it is unlikely to be useful for detecting uncharacterized pathogens. An appropriate target should either be pathogen-specific (like *pagA* and Spike E) or exhibit enough sequence variation between species (like *rpoB*) to avoid falsely detecting closely related species. Target transcript abundance is also an important consideration, as highly expressed genes are superior targets for HC assays due to a higher target-to-cellular RNA ratio in samples, resulting in a lower LOD. Transcript length also plays a crucial role in determining the assay’s sensitivity, as demonstrated by the differences in maximum signal and LOD among *atxA*, *gyrA*, *pagA* and *rpoB* hybrids; longer hybrids with more S9.6 epitopes per molecule have a lower LOD than shorter hybrids.

Multiplexed probes targeting multiple genes specific to the pathogen have the potential for enhancing the assay's sensitivity. To assay for multiple pathogen-specific target transcripts using full-length probes would generate signals from a greater proportion of the cellular RNA, while using multiple small detection probes combined with a capture probe could overcome reduced hybridization efficiency caused by secondary structures in long RNA transcripts. The HC-ELISA assay is simple and can be adopted into sentinel labs, reference labs and national laboratories for pathogen detection to detect active infection at low cost. As this manuscript was prepared for submission, new reports on the use of S9.6 in diagnostics emerged, supporting our findings that it can be adopted for detecting any gene or transcript specific to a disease or pathogen^13–15^. Considering the usage of non-amplification-based nucleic acid detection for pathogen-specific targets, we find our approach to be 10 to 100-fold more sensitive in detecting in-vitro synthesized transcripts than the assay developed by Dey et al.^13^. However, the introduction of transcription-mediated-isothermal-RNA-amplification by Dey *et. al*. does improve the assay sensitivity to LOD = 10 copies/µl. Additionally, a different approach using primer exchange reaction-amplified protein-nucleic acid interactions could identify small DNA-RNA hybrids in micro-RNAs at a range of 100 aM, corresponding to 10^3^ copies/sample^14^.

## Conclusion

We developed a DNA-RNA Hybrid Capture immunoassay (HC) capable of detecting pathogen RNA transcripts using the S9.6 antibody, which specifically recognizes DNA-RNA hybrids in a sequence-independent manner. We developed the HC method in an ELISA format (HC-ELISA) and adapted it to a lateral flow format (HC-LFA). HC-ELISA has demonstrated the sensitivity to detect as few as 10^4^ molecules of 2.2 kb-long RNA transcripts, while HC-LFA has shown the ability to identify the presence of *B. anthracis* equivalent to a bacterial load of 10^7^ CFU. We successfully detected *B. anthracis rpoB*, *pagA*, and *gyrA* RNA from in vitro cultures' cellular RNA extracts and *rpoB* from simulated and real infections in mouse spleen with a total turnaround time of ~ 5 h. Moreover, we showcased HC's pan-pathogen adaptability by detecting B1 gene RNA from a simulated *T. gondii* infection and Spike E gene RNA of SARS-CoV-2 from in vitro transcription. Consequently, HC-ELISA and HC-LFA can detect the presence of long RNA transcripts (> 1.5 kb) from any organism without molecular manipulations such as reverse transcription or nucleic acid amplification. Future work could explore utilizing multiple pathogen-specific probes to further enhance detection limits.

## Materials and methods

### Plasmids and bacteria

Plasmids and bacterial strains used for this study are listed in Table 1. The SARS-CoV-2 gene Spike E was obtained from plasmid pET30a-Spike-E, which was a gift from Rick Tarleton (Addgene plasmid # 165096)^42^. *B. anthracis pagA* gene plasmid pET15b-His-PA was created in this laboratory. The B1 gene of *T. gondii* is a 1384 bp-long gene with 35 tandem copies from head to tail, harboring an intron of 388 bp. To clone the B1 gene without introns for the purpose of probe preparation, the fragments upstream (F1) and downstream (F2) of the intron were PCR amplified using the primers listed in Table 2. The amplified F1 and F2 fragments of the B1 gene were cloned into the pJET1.2 blunt end vector from the CloneJET PCR Cloning Kit (Thermo Fisher Scientific) following the NEBuilder HiFi DNA Assembly Kit protocol (New England Biolabs [NEB]). The non-encapsulated Sterne-like *B. anthracis* A35 strain^43^ was grown in NBY media at 37 °C to A_650_ of ~ 1.0 prior to harvest. Growth conditions for some studies included 0.8% NaHCO_3_ and 5% atmospheric CO_2_. Spores used in mouse infections were prepared as previously described^41^.

**Table 1:** List of plasmids and strains used in this study.

Plasmid and bacterial strains	Relevant characteristics	References
pET15b-His-PA	*pagA* gene under T7 promoter	Lab collection
pJET-B1	*T. gondii* B1 locus (intron removed) under T7 promoter	This study
pET30a-Spike-E	SARS-CoV-2 Spike E gene under T7 promoter	^42^

**Table 2:** List of primers and oligonucleotides.

Primer	Sequence (5′–3′)	Reference
atxA_Rev_Bio	/5Biosg/ATTCATCATAATTTATCCCA	This study
atxA_For_5P	/5′Phos/ATGCTAACACCGATATCCATC	This study
pagA_Rev_Bio	/5′Biosg/TCCTATCTCATAGCCTTTTT	This study
pagA_For_5P	/5′Phos/ATGAAAAAACGAAAAGTGTTAATAC	This study
gyrA_Rev_Bio	/5′Biosg/TTATTCTTCTGAAGAAACCTCTTCGCT	This study
gyrA_For_5P	/5′Phos/ATGTCAGACAATCAACAACAAGCACG	This study
rpoB_Rev_5Bio	/5′Biosg/TTATTCCTTAGTTGTCTCAAC	This study
rpoB_For_5P	/5′Phos/TTGACAGGTCAACTAGTTCAATACG	This study
Spike E_Rev_Bio	/5′Biosg/TGCTCGAGTTAACCCGGACCTTGAAACAGA	This study
Spike E_For_5P	/5′Phos/CTGGGTACCATGTTCGTTTTCCTGGTTCTG	This study
B1_For_5P	/5′Phos/GAATTCGTTCGACAGAAAGGGAGCAAG	This study
B1_1.3k_Rev_Bio	/5Biosg/GAGACGAACACGCTAGAGCAGTTGTTGC	This study
pagA_RT_F	AATGAATCAGAATCAAGTTCC	This study
pagA_RT_R	ATGTATATTCATCACTCTTCTTAAC	This study
rpoB_RT_F	ACTGCTACTGTAATTCCAAACCGCGG	This study
rpoB_RT_R	AAACCCTAATGCGCGTAACAAAACAG	This study
gyrA_RT_F	CCTCATGGTGATTCAGCTGT	This study
gyrA_RT_R	AGATCCAAAGTTACCATGCC	This study
B1_F1_F	CTCGAGTTTTTCAGCAAGATGAATTCGTTCGACAGAAAG	This study
B1_F1_R	GGCGACCAATCCTTCTTCTGGCATTTGC	This study
B1_F2_F	CAGAAGAAGGATTGGTCGCCTGCAATCG	This study
B1_F2_R	AGGAGATCTTCTAGAAAGATGAGACGAACACGCTAGAG	This study

### Toxoplasma

Tachyzoites from type II Prugniaud (luciferase-expressing) *T. gondii* parasites^44^ were grown in Human foreskin fibroblast (HFF) cells following standard protocols^45^. Briefly, cells were grown in DMEM media supplemented with 10% fetal bovine serum, 2 mM GlutaMAX and 1 mM sodium pyruvate at 37 °C with 5% CO_2_ to 70–80% confluence in T-25 cells prior to infection with various doses of parasite.

For harvest, cells were scraped from flasks showing different levels of parasite-induced lysis as assessed by monolayer destruction (40–90%). This material was passed through a syringe (27-gauge needle) once and centrifuged at 300 rpm for 5 min to remove bulk of cell debris. From each T-25 flask, half of the post-spin supernatant containing tachyzoites was centrifuged at a higher speed (1300 rpm, 10 min) and pellets resuspended in 1 mL of TRIzol (Invitrogen) followed by processing as described for *B. anthracis* samples.

### Mice

C57BL/6 J mice (in-house, male, 8 weeks old) were used as a source of uninfected spleens or in spore challenge studies. Mice were subcutaneously (SC) infected with 2 × 10^7^ *B. anthracis* A35 spores prepared in PBS (1 mL) in the scruff of neck and monitored daily for signs of malaise prior to euthanasia and spleen harvest. All animal experiments were performed in strict accordance with guidelines from the NIH and the Animal Welfare Act, under protocols approved by the Animal Care and Use Committee of the National Institute of Allergy and Infectious Diseases, National Institutes of Health (protocol LPD9E).

### Cellular RNA isolation

Cellular RNA from bacteria, protozoan, and mouse spleen was isolated using TRIzol (Invitrogen). 50–100 mg wet-weight *B. anthracis* culture was resuspended in 1 mL TRIzol and 250 µL of 0.1 mm zirconia beads was added (BioSpec Products). The cells were homogenized at 6.0 m/s speed for 60 s using FastPrep-24 (MP Biomedicals) then incubated on ice for 5 min. Following three rounds of homogenization, RNA was isolated according to the manufacturer’s protocol and used directly as cellular RNA for dot blot and hybrid capture assays. For C57BL/6J mouse spleen, a single cell suspension was made from 100 mg of organ material filtered through a 70 µm cell strainer (BD Falcon) and combined with TRIzol (1 mL) prior to RNA extraction. *B. anthracis* vegetative bacteria were spiked in select samples at specific doses in some studies.

### In vitro transcription

Plasmids carrying the *B. anthracis pagA* and SARS-CoV-2 Spike E genes were used as templates for transcription following linearization (which improved the reaction’s quality and yield) with FastDigest BamHI for pET15b-His-PA or FastDigest XhoI for pET30a-Spike-E (Thermo Scientific). In vitro transcription was performed using T7 RNA Polymerase according to the manufacturer’s protocol (NEB). The reaction was then treated with TURBO DNase I (Invitrogen) and purified using the RNeasy Mini Kit (Qiagen). Generally, 500 ng of plasmid yielded ~ 50 ng of in vitro-transcribed RNA.

### Single-stranded DNA (ssDNA) probe synthesis

Full length individual genes were amplified by PCR using LongAmp Taq DNA polymerase (NEB), equimolar concentrations of 5′-phosphorylated forward primer and 5′-biotinylated reverse primer (Table 2), and 0, 1, or 10 µM biotin-14-dATP (Invitrogen) (Fig. S1C–G). Probes synthesized without added biotin-14-dATP are termed “P_M_” for “mono-biotinylated probe”, while those synthesized with added biotin-14-dATP are termed “P_1_” and “P_10_” for “1/10 µM poly-biotinylated probe”, respectively. Lambda Exonuclease (NEB) was used to digest the 5′-phosphorylated sense strand leaving behind a biotinylated ssDNA probe. In general, 4 µL enzyme could digest 750 ng PCR product in a 50 µL reaction over 3 h at 37 °C. The ssDNA band was excised from 1% agarose gel and purified using Zymoclean Gel DNA Recovery Kit (Zymo Research). Probe purity was confirmed with Thermolabile Exonuclease I treatment (NEB).

### Expression and purification of S9.6 monoclonal IgG

S9.6 IgG expression and purification protocols were followed as previously reported^20^. S9.6 IgG-producing hybridoma cells (HB-8730) were grown in DMEM supplemented with 10% fetal bovine serum, 2 mM GlutaMAX, 1 mM sodium pyruvate, and 50 μg/mL gentamycin (Life Technologies, Carlsbad, CA). The S9.6 IgG was purified from culture supernatants using HiTrap Protein G columns (GE Healthcare Life Sciences, Piscataway, NJ).

### Nucleic acid hybrid formation

DNA-RNA hybrids were generated from respective ssDNA probes and in vitro transcribed mRNA or cellular RNA. For in vitro transcribed mRNA, 1.25 ng of respective DNA probe was mixed with an equal amount of mRNA in a hybridization buffer (25 mM Tris–Cl, pH 8.0, 150 mM NaCl, 1 mM MgCl_2_). For cellular RNA, 5 ng of probe (unless mentioned; based on calculations from Ct values in qPCR) was mixed with 2 µg of cellular RNA. To achieve hybridization, the reaction was heated at 95 °C for 3 min and transferred to a boiling water bath covered with aluminum foil, which was allowed to gradually cool to room temperature. Alternatively, hybridization was achieved using a thermocycler programmed to hold sample temperature at 95 °C for 3 min, then cool 0.1 °C every 6 s to the equivalent of room temperature.

### Nuclease treatment

For the dot blot assay, cellular RNA from *B. anthracis* was treated with nucleases of various specificities including RNase H, ShortCut RNase III, and Mung Bean Nuclease (NEB); RNase T1 and TURBO DNase I (Invitrogen); and RNase A (Sigma-Aldrich). The treatments were carried out following the manufacturer’s protocols, with incubation at 30 °C for Mung Bean Nuclease and 37 °C for all other nucleases, prior to spotting on the membrane. For HC-ELISA, nucleic acids were treated after hybridization with the nucleases listed above, except Mung Bean Nuclease. Enzyme treatment was performed according to each manufacturer’s protocol with the following quantities of enzyme used: RNase H, 0.2 uL (1 U); RNase III, 1 uL (2 U); Mung Bean Nuclease, 0.5 uL (5 U); RNase T1, 1 uL (1000 U); DNase I, 0.5 uL (1 U); RNase A, 1 uL (1.5 mU).

### Dot blot assay

A 200 µl of DNA-RNA hybrids and related controls were treated with nucleases (described above) and transferred in 20× SSC by vacuum manifold (Merck Millipore) onto a Nylon H + Hybond membrane (Amersham, Merck). Nucleic acids were crosslinked to the membrane by baking (2 h, 80 °C) and the membrane was then rehydrated in 20X SSC buffer prior to blocking with Intercept TBS Blocking Buffer (LiCOR; 1 h) and adding S9.6 (1 µg/mL, 16 h, 4 °C). The membrane was washed 3 times with 1X TBS + 0.05% Tween-20 prior to incubation with DyLight 800 dye conjugated anti-mouse IgG (Rockland; 1:10,000, 1 h). The membrane was again washed 3 times and imaged using Odyssey CLx (LiCOR). Pixel intensity for each sample on the dot blot was calculated using Image Studio software (LiCOR).

### Quantitative real-time polymerase chain reaction (qPCR)

Cellular RNA was treated with TURBO DNase I and purified using RNeasy Mini Kit; 1 µg purified RNA was used to synthesize cDNA with iScript™ cDNA Synthesis Kit (Bio-Rad). qPCR was performed with 5 ng of cDNA template per reaction in the QuantStudio™ 7 Flex Real-Time PCR System (Thermo Fisher Scientific).

### Hybrid capture immunoassay by ELISA (HC-ELISA)

For DNA-RNA detection by a hybrid capture ELISA format (HC-ELISA), DNA-RNA hybrids containing biotinylated DNA were captured onto streptavidin-coated clear 96-well plates (Pierce, Invitrogen) in binding buffer (50 mM TrisCl, pH 7.5, 150 mM NaCl, 0.005% Tween 20, 0.1% BSA) at room temperature for 2 h. Post-binding, the plate was washed 3 times with wash buffer (25 mM TrisCl, pH 7.5, 150 mM NaCl, 0.1% Tween 20, 0.1% BSA). S9.6 in Intercept TBS Blocking Buffer was added to the plate (1 µg/mL, 16 h, 4 °C). Three washes were repeated prior to incubation with HRP-conjugated chicken anti-mouse antibody (Aves Labs; 1:10,000, 1 h) and development with SuperSignal ELISA Femto Substrate (Thermo Fisher Scientific), reading signal on a Victor3V microplate reader (PerkinElmer).

### DNA-RNA hybrid-capture immunoassay by lateral flow assay (HC-LFA)

Lateral flow strips, chase buffer and S9.6 gold nanoparticle conjugates (S9.6-GNP) were used in HC-LFA using the proprietary knowledge of DDTD, CA. BioReady 150 nm gold nanoshells functionalized with carboxylic acid groups (nanoComposix) were used to conjugate S9.6 IgG, using manufacturer’s protocol. The control (C) and test (T) lines on the nitrocellulose lateral flow strips were prepared using solutions of 1 mg/mL of goat anti-mouse IgG (Lampire Biological Laboratories, Pipersville, PA) and 0.5 mg/mL of streptavidin (Sigma-Aldrich), respectively. Nucleic acids were added to the sample port in volumes up to 10 µL followed by sequential addition of 2–4 µL S9.6-GNP and ~ 100 µL of chase buffer (2–3 drops) to the conjugate port. The strip developed for 30 min prior to imaging and analysis on a Leelu Reader (Lumos Diagnostics, Carlsbad, CA).

### Supplementary Information

Supplementary Information.

## Data Availability

The authors declare that: the datasets used and/or analyzed during the current study available from the corresponding author on reasonable request. All data generated or analyzed during this study are included in this published article [and its supplementary information files].

## Abbreviations

LFA: Lateral flow assay
NATs: Nucleic acid tests
HC: Hybrid capture immunoassay (HC)
dsRNA: Double stranded RNA
ssRNA: Single stranded RNA
LOD: Limit of detection
P_M_: 5′-Mono-biotinylated DNA probe
P_1_: Poly-biotinylated DNA probe using 1 µM biotin-14-dATP
P_10_: Poly-biotinylated DNA probe using 10 µM biotin-14-dATP
GNP: Gold nanoparticle
