# Supplementary material for: S9.6-based hybrid capture immunoassay for pathogen detection
Source: Sci Rep. 2023 Dec 19;13:22562. doi: 10.1038/s41598-023-49881-w (PMC10728093; doi:10.1038/s41598-023-49881-w)
Supplement: Supplementary file 1 — Supplementary Information. [file 41598_2023_49881_MOESM1_ESM.docx]

**Supporting Information**

**S9.6-based hybrid capture immunoassay for pathogen detection**

Ankur Bothra^1^*, Megan L. Perry^1^, Elena Wei^1^, Mahtab Moayeri^1^, Qian Ma^1^, Marco A. Biamonte^2^, Marina Siirin^2^, Stephen H. Leppla^1^,

^1^Microbial Pathogenesis Section, Laboratory of Parasitic Diseases, National Institute of Allergy and Infectious Diseases, Bethesda, MD

^2^Drugs & Diagnostics for Tropical Diseases, San Diego, CA

*Correspondence to Ankur Bothra ([ankur.bothra@nih.gov](mailto:ankur.bothra@nih.gov))

**Figure S1.** Complete dot blot image from Figure 1 and ssDNA probe preparation scheme including representative images of agarose gels displaying probes.

**Figure S2.** Predicted secondary structure of *pagA* mRNA, signal-to-noise heatmap of *rpoB* HC-ELISA shown in Figure 2C, signal intensity of HC-ELISA by transcript length.

**Figure S3.** Efficiency of HC-ELISA to detect simulated infection as compared to detection of bacteria alone.

**Figure S4.** Supplementary data for HC-LFA performance in simulated and live infections of mice.

**Table S1.** Summary of mouse anthrax infections and HC-LFA results presented in Figure S4.

**Full length blot and gel images for editorial purpose.**

**
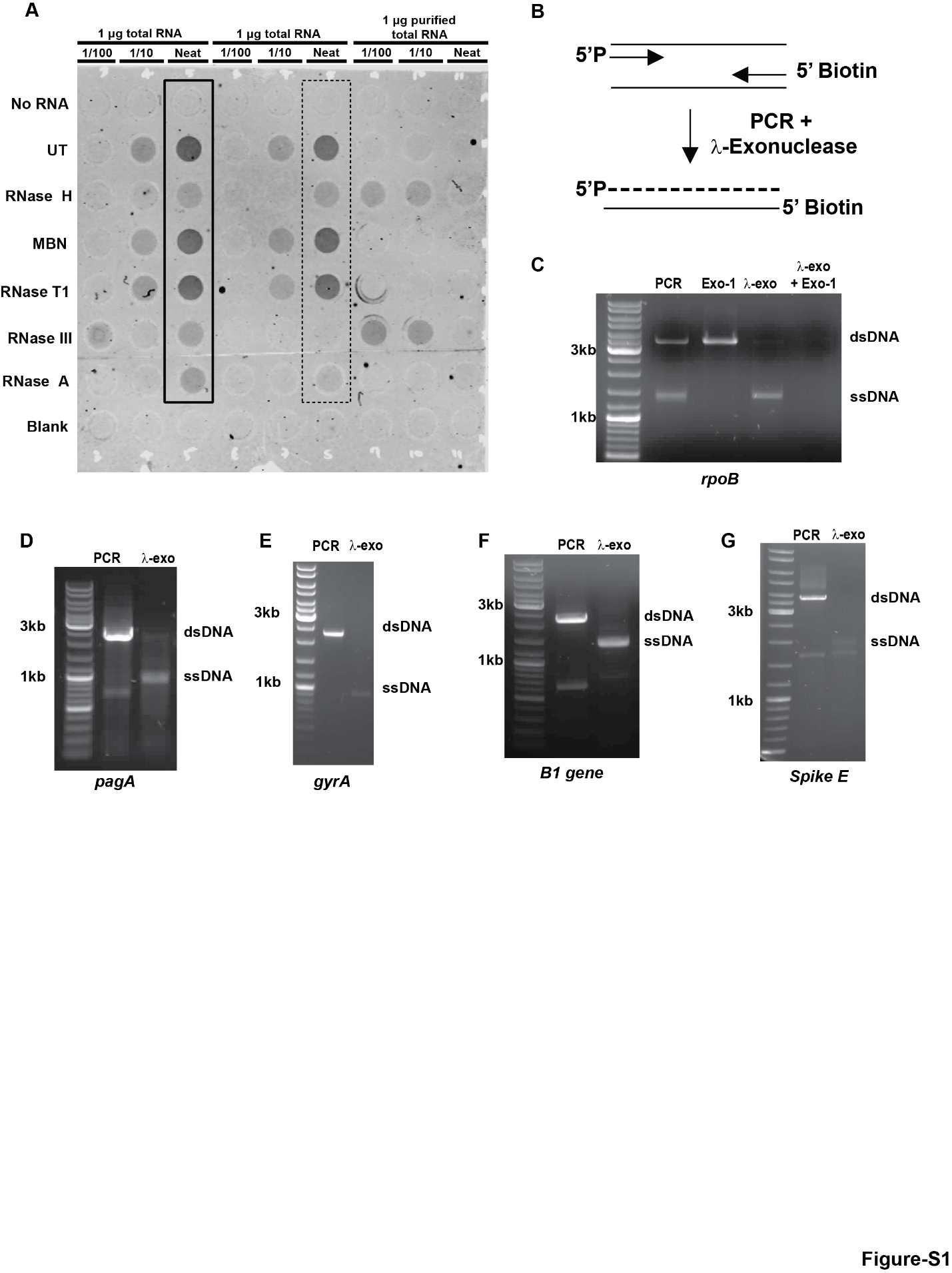
**

**Figure S1.** (A) Complete image of dot blot from data presented in Figure 1A-B. The solid line box contains the image presented in 1A; the dotted line box contains dots used for pixel intensity quantification in 1B in addition to the image presented in 1A. (B) Strategy for DNA probe synthesis as described in Materials and Methods. (C-G) Representative agarose gel electrophoresis analyses including PCR amplicon and single-stranded lambda exonuclease product; (C-E) *B. anthracis* genes (C) *rpoB* with ssDNA identity tested using thermolabile exonuclease-1 (Exo-1), (D) *pagA*, (E) *gyrA*; (F) *T. gondii* B1 gene; (G) SARS-CoV-2 Spike E gene.


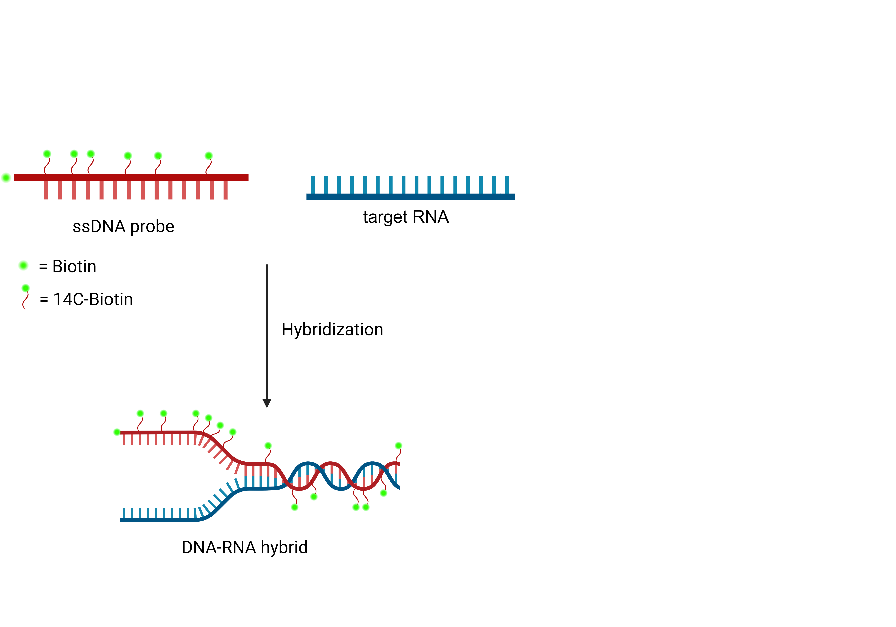
**
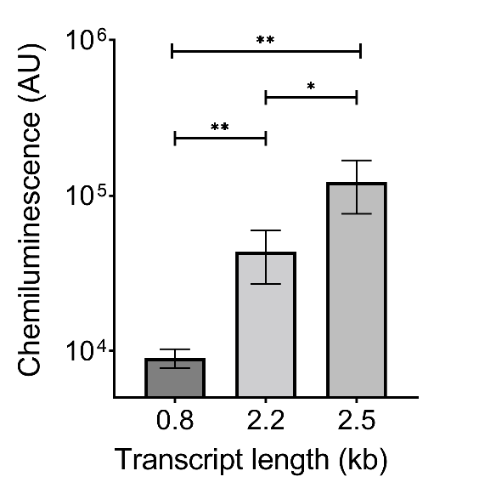

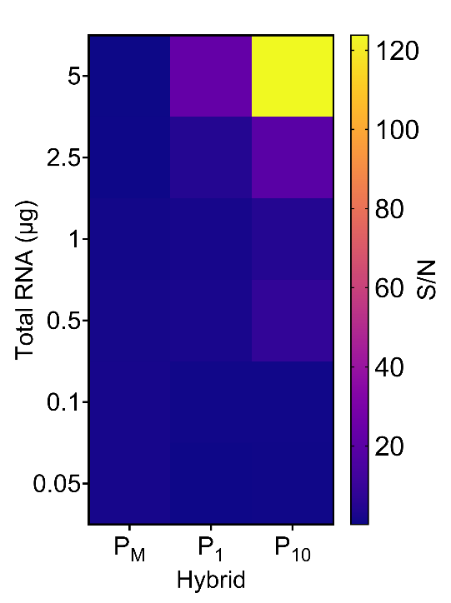

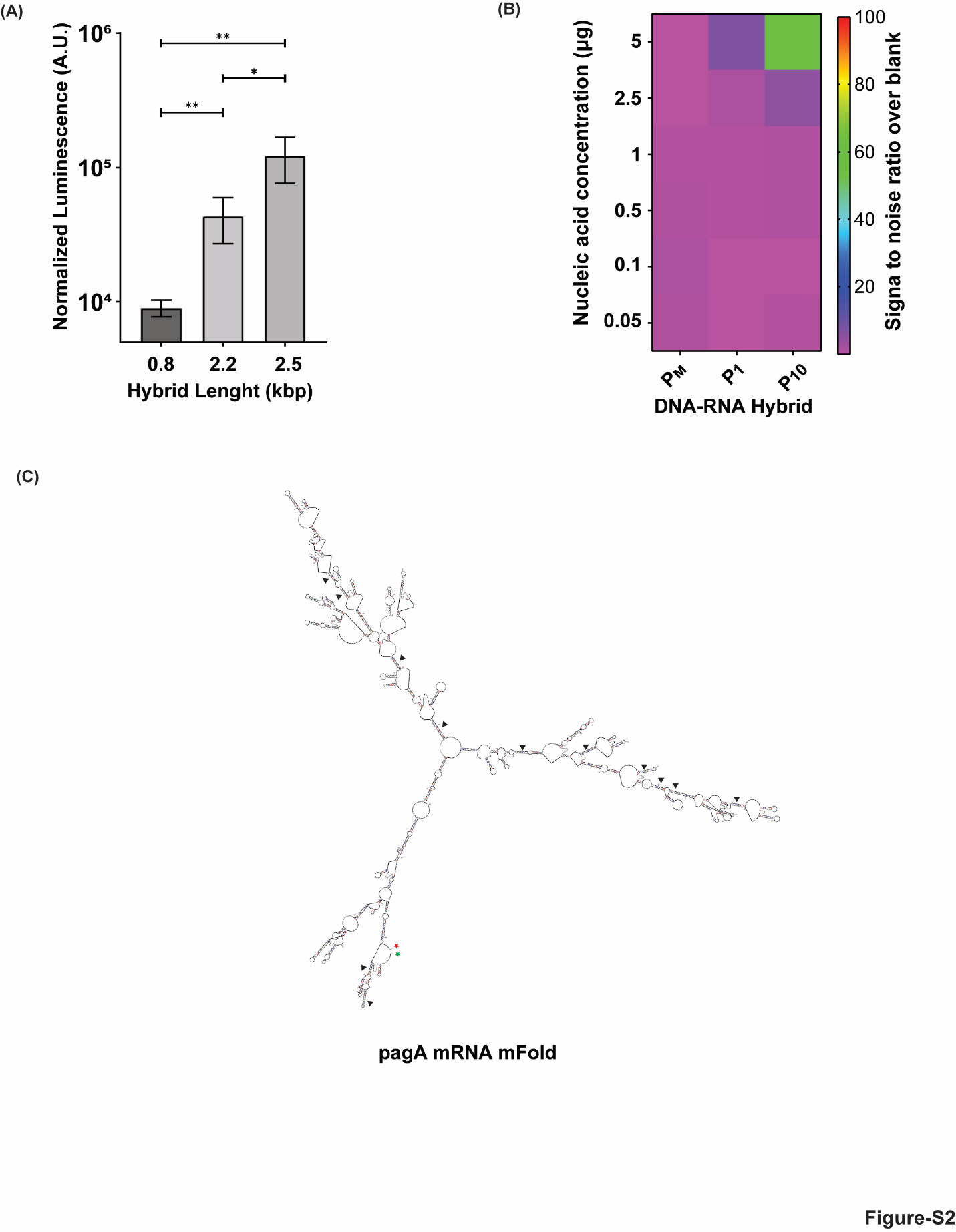
**

**D**

**C**

**B)**

**A**

**Figure S2.** (A) Predicted secondary structure of *pagA* mRNA using mFold server. (B) Possible hybridization of biotinylated-ssDNA (probe) to its target RNA resulting in the formation of DNA-RNA hybrids. (C) HC-ELISA signal intensity for transcripts of length 0.8 kb (*atxA*), 2.2 kb (*pagA*), and 2.5 kb (*gyrA*) from *B. anthracis* total RNA. (D) Signal to noise ratio (S/N) as signal observed from hybrid over signal from RNA alone calculated for the data from Figure 2C.


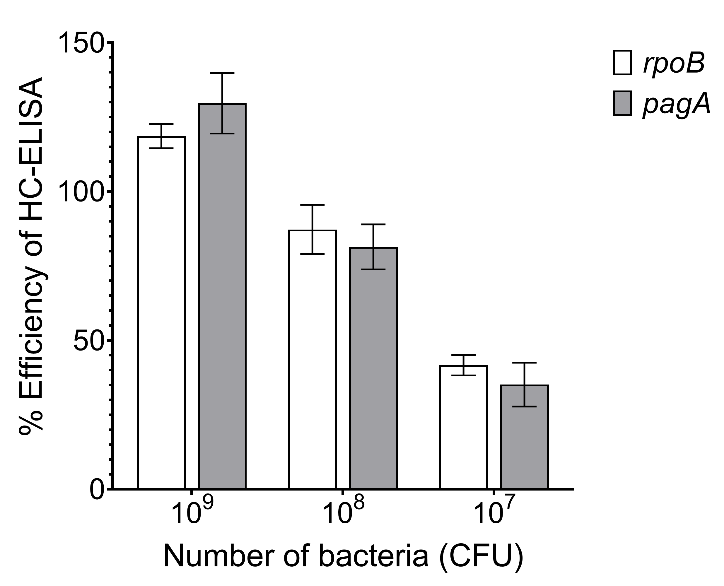


**Figure S3.** Efficiency of HC-ELISA to detect *rpoB* and *pagA* in mouse spleen spiked with *B. anthracis* as percent of signal obtained from equivalent bacterial loads of *B. anthracis* alone. Data represents mean ± SD of triplicate wells in a representative experiment of n = 3.

**Figure S4.** (A) Quantification of lateral flow images in Figure 4C. Data represents the line area intensity of test over control captured once for each strip at 20 and 30 minutes. (B) Images of HC-LFA targeting *rpoB* in total RNA extracted from the spleen of C57BL/6J mice infected with *B. anthracis* spores (2×10^7^ A35, SC)*.*

**Table S1.** Spleen samples from *B. anthracis* spore-infected mice for which HC-LFA was performed in Figure S4.

| **Sample #** | **# of spleens** | **Malaise grade(s)** | **HC-LFA** |
| --- | --- | --- | --- |
| 1 | 2 | 2, 2.5 | ++ |
| 2 | 1 | 2.5 | +++ |
| 3 | 1 | 3 | +++ |
| 4 | 2 | 1.5, 2 | - |
| 5 | 1 | 3 | + |

**Ful length blot and gel images:**

For figure-1A: Complete image of dot blot from data presented in Figure 1A-B. The solid line box contains the image presented in 1A; the dotted line box contains dots used for pixel intensity quantification in 1B in addition to the image presented in 1A.

For figure-1B: Duplicate samples of RNA alone (1, 1’), ssDNA alone (2, 2’) and Hybrids (3, 3’) were tested on Dot-blot.


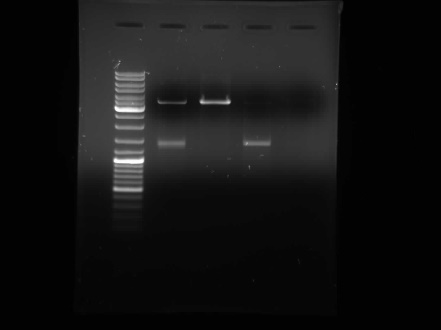


For figure S1C: Complete image of ssDNA probe from data presented in Figure S1C


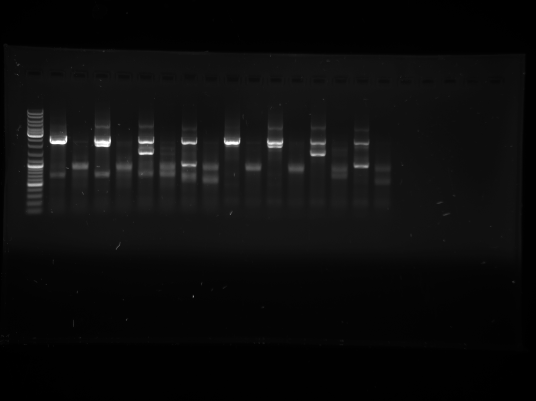


For figure S1D: Complete image of ssDNA probe from data presented in Figure S1D. Marked area was represented in Supplementary figure S1D. Other lanes include PCR and respective λ-exo treated *pagA* ssDNA produced using gradient PCR.


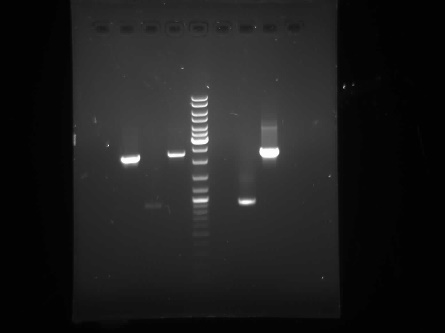


For figure S1E: Complete image of ssDNA probe from data presented in Figure S1E. Marked area was represented in Supplementary figure S1E. Other lanes are not relevant for *gyrA* ssDNA probe preparation and its respective λ-exo treatment.


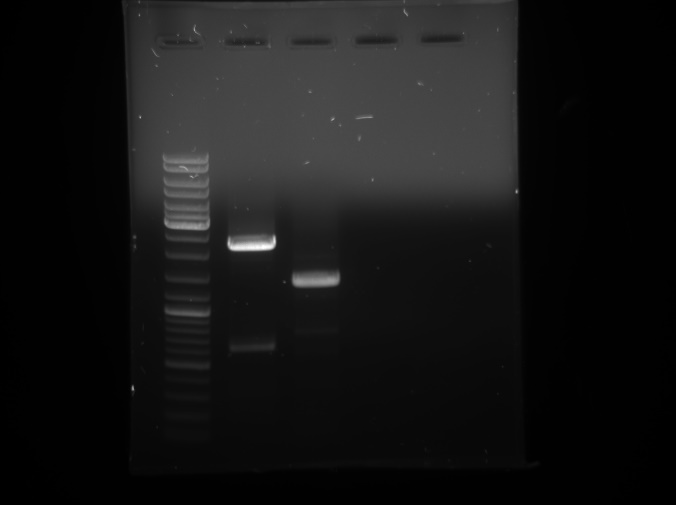


For figure S1F: Complete image of ssDNA probe from data presented in Figure S1F.


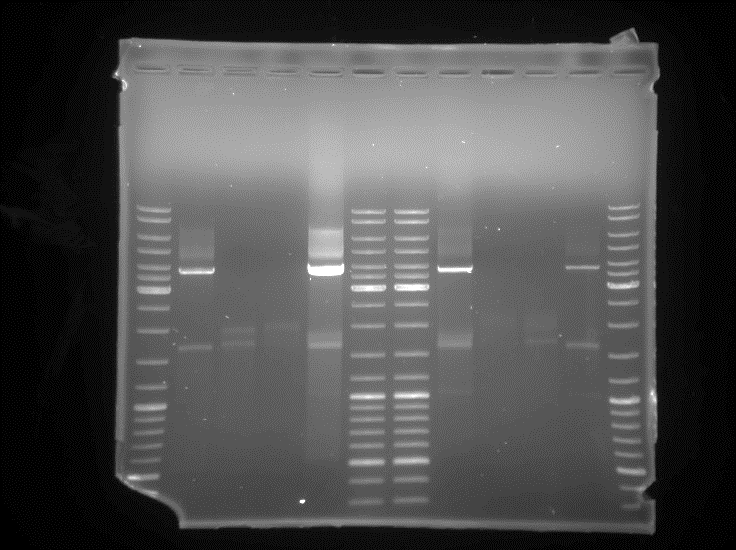


For figure S1G: Complete image of ssDNA probe from data presented in Figure S1G. Marked area was represented in Supplementary figure S1G. Other lanes include PCR and respective λ-exo treated *spikeE* ssDNA produced using gradient PCR.
